# Evaluation of the anterior acetabular coverage with a false profile radiograph considering appropriate range of positioning

**DOI:** 10.1038/s41598-023-35514-9

**Published:** 2023-05-22

**Authors:** Yasuhiko Kokubu, Shinya Kawahara, Kenji Kitamura, Satoshi Hamai, Goro Motomura, Satoshi Ikemura, Taishi Sato, Ryosuke Yamaguchi, Daisuke Hara, Masanori Fujii, Yasuharu Nakashima

**Affiliations:** 1 Department of Orthopaedic Surgery, Graduate School of Medical Sciences, Kyushu University, 3-1-1 Maidashi, Higashi-Ku, Fukuoka, 812-8582 Japan; 2 Department of Medical-Engineering Collaboration for Healthy Longevity, Kyushu University, 3-1-1 Maidashi, Higashi-Ku, Fukuoka, 812-8582 Japan; 3 Department of Orthopaedic Surgery, Faculty of Medicine, Saga University, 5-1-1 Nabeshima, Saga, 849-8501 Japan

**Keywords:** Anatomy, Medical research

## Abstract

This study aimed to (1) set a reference value for anterior center edge angle (ACEA) for preoperative planning of periacetabular osteotomy (PAO), (2) investigate the effects of pelvic rotation and inclination from false profile (FP) radiographs on the measured ACEA, and (3) determine the “appropriate range of positioning” for FP radiograph. This single-centered, retrospective study analyzed 61 patients (61 hips) who underwent PAO from April 2018 and May 2021. ACEA was measured in each digitally reconstructed radiography (DRR) image of the FP radiograph reconstructed in different degrees of pelvic rotation. Detailed simulations were performed to determine the “appropriate range of positioning” (0.67 < ratio of the distance between the femoral heads to the diameter of the femoral head < 1.0). The vertical-center-anterior (VCA) angle was measured on the CT sagittal plane considering the patient-specific standing positions, and its correlation with the ACEA was investigated. The reference value of ACEA was determined by receiver operating characteristic (ROC) curve analysis. The ACEA measurement increased by 0.35° for every 1° pelvic rotation approaching the true lateral view. The pelvic rotation with the “appropriate range of positioning” was found at 5.0° (63.3–68.3°). The ACEA on the FP radiographs showed a good correlation with the VCA angle. The ROC curve revealed that an ACEA < 13.6° was associated with inadequate anterior coverage (VCA < 32°). Our findings suggest that during preoperative PAO planning, an ACEA < 13.6° on FP radiographs indicates insufficient anterior acetabular coverage. Images with the “appropriate positioning” can also have a measurement error of 1.7° due to the pelvic rotation.

## Introduction

Periacetabular osteotomy (PAO) is a valid surgical option for treating symptomatic developmental dysplasia of the hip (DDH)^1–7^. This procedure can be used to correct acetabular coverage three-dimensionally and prevent or delay osteoarthritis progression^2,3^. Many studies have shown that correcting the lateral acetabular coverage can affect the longevity of the native hip following PAO^6,7^. However, recent studies have shown that postoperative anterior acetabular coverage has a greater impact on postoperative hip longevity^4,5^. Furthermore, weight-bearing radiographs are more appropriate for evaluating hip deformity because DDH causes abnormal mechanical loading in the weight-bearing position rather than in the supine position^8–10^. Additionally, patient-specific pelvic inclination should be considered because radiographic measurements are significantly affected by changes in pelvic inclination between the standing and supine positions^8,11^.

Kitamura et al. performed virtual PAO and reported that anterior acetabular rotation should be considered in addition to lateral rotation in PAO with a preoperative anterior acetabular coverage angle of < 32° in the CT sagittal plane in the patient-specific standing position^12^. CT is a useful tool to study pelvic morphology in detail, but routine imaging is not performed in all institutions because of the high radiation dose and cost^13^. In addition, understanding the three-dimensional anatomic features from the two-dimensional radiographs is essential for correcting the acetabular coverage three-dimensionally.

**Figure 1:**
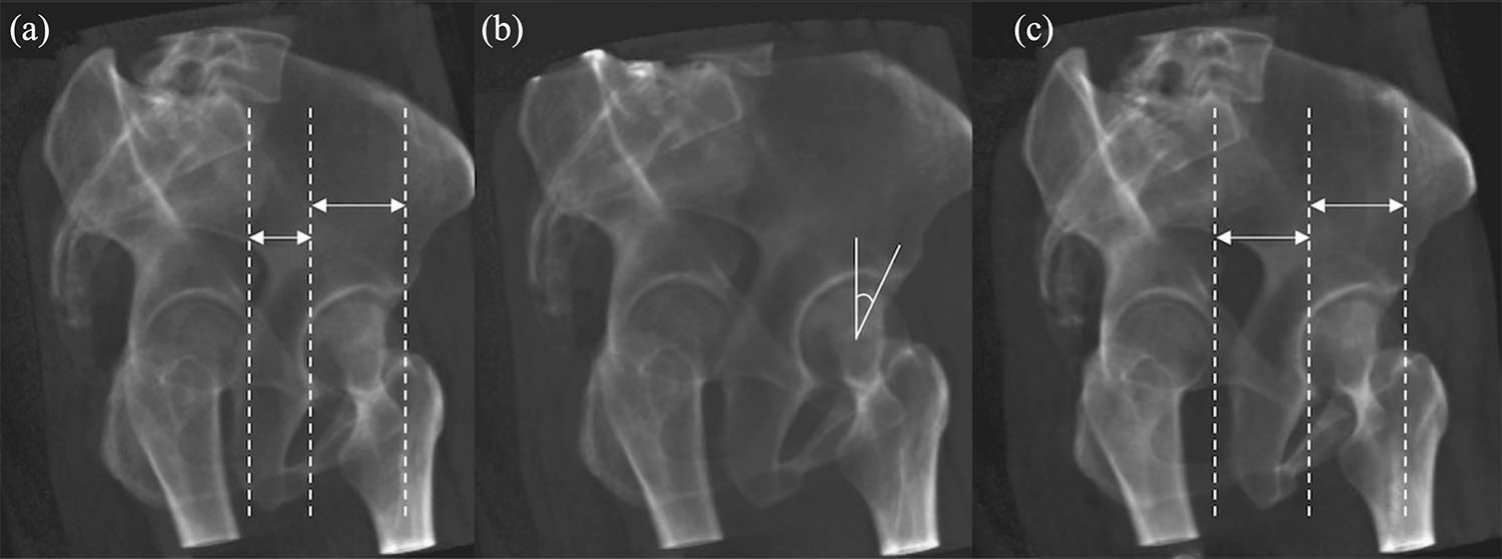
FP radiographs of different pelvic rotations are shown in (**a**–**c**). ACEA is measured in a FP radiograph at 65°of rotation (**b**). Investigated the “appropriate range of positioning” where the distance-to-diameter ratio is between 0.67 (a: 68.1°of rotation) and 1.0 (c: 63.2°of rotation). The arrows indicate the distance between the femoral heads and the femoral head diameter. *ACEA* Anterior center edge angle, *FP* False profile.

The anterior center edge angle (ACEA) is a commonly used radiographic parameter for evaluating anterior acetabular coverage^14,15^. The ACEA is the projected angle from the anterolateral margin of the acetabulum as measured using a false profile (FP) radiograph. However, the ACEA has not been used as a valuable parameter for preoperative planning, and there is no clear reference value. The reason is that radiographic measurements are affected by the pelvic position^16–18^. As originally reported by Lequesne and de Size^15^, the following criterion for “appropriate range of positioning” has been used widely: the horizontal distance between the medial aspects of the femoral heads was between 67 and 100% of the femoral head **(**Fig. 1**).** However, few studies have examined the validity of this criterion in detail^17^. Moreover, there is a need to understand the extent of the measurement error that will occur within the range of rotation that is judged to be appropriate.

Therefore, this present study had the following objectives: to use three-dimensional (3D) computer simulations to (1) set a valid reference value for the preoperative planning of PAO, (2) investigate the effects of pelvic rotation and inclination from FP radiographs on the measured ACEA, and (3) determine the “appropriate range of positioning”.

## Methods

### Patient selection and data acquisition

**Figure 2:**
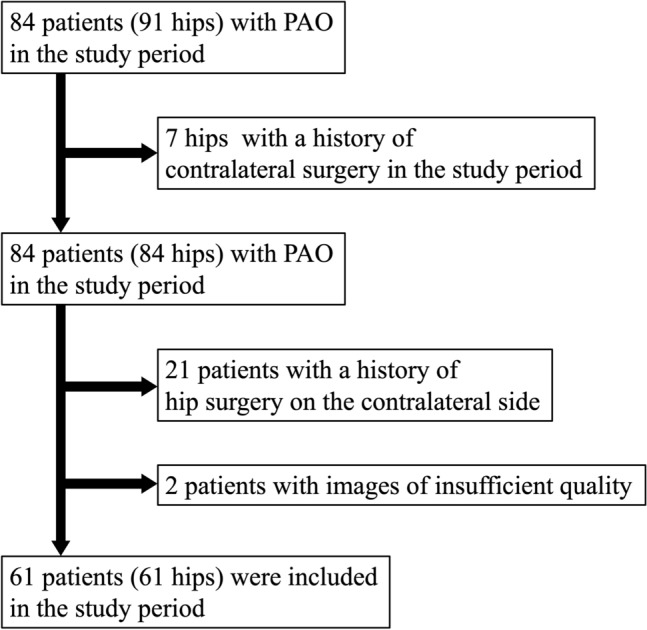
The Strengthening the Reporting of Observational Studies in Epidemiology (STROBE) diagram for inclusion process. *PAO* Periacetabular osteotomy.

This is a case series with a level of evidence of 4. Eighty-four consecutive patients (91 hips) who underwent PAO for symptomatic DDH between April 2018 and May 2021 were included in this study. All of the patients were Japanese. The indications for PAO included both clinical symptoms (hip pain that interfered with daily activities) and radiological evidence of DDH (a lateral center–edge angle [LCEA] of < 20°, a Tönnis angle of > 10°, and a sharp angle of > 45°^19–22^) or borderline dysplasia (defined by an LCEA between 20° and 25°^21,22^). In seven patients with bilateral PAO during this period, only the first operated side was included. Twenty-one patients were excluded because they had a history of hip surgery on the contralateral side. Two patients with images of insufficient quality for analysis were also excluded. After eligibility assessment, 61 patients (61 hips) were enrolled in this study (Fig. 2).

Supine and standing anteroposterior (AP) pelvic radiographs and CT images were obtained during preoperative planning of PAO. Preoperative transverse CT scans (Aquilion ONE; Canon Medical Systems Corporation, Tochigi, Japan) of the whole pelvis and femur (from the top of the iliac crest to the distal femur) were obtained in all patients with a 512 × 512 image matrix, a 0.35 × 0.35 pixel dim, and a 1-mm thickness. The patients were placed supine on a scanning table and instructed to naturally extend their affected hip and knee joints. The CT images were acquired using digital imaging and communications in medicine (DICOM) format data from the CT system server. The supine and standing radiographs were investigated using Fujifilm OP-A software (Fujifilm, Co., Ltd, Tokyo, Japan).

### Definition of the pelvic 3D coordinate system in the standing position

**Figure 3:**
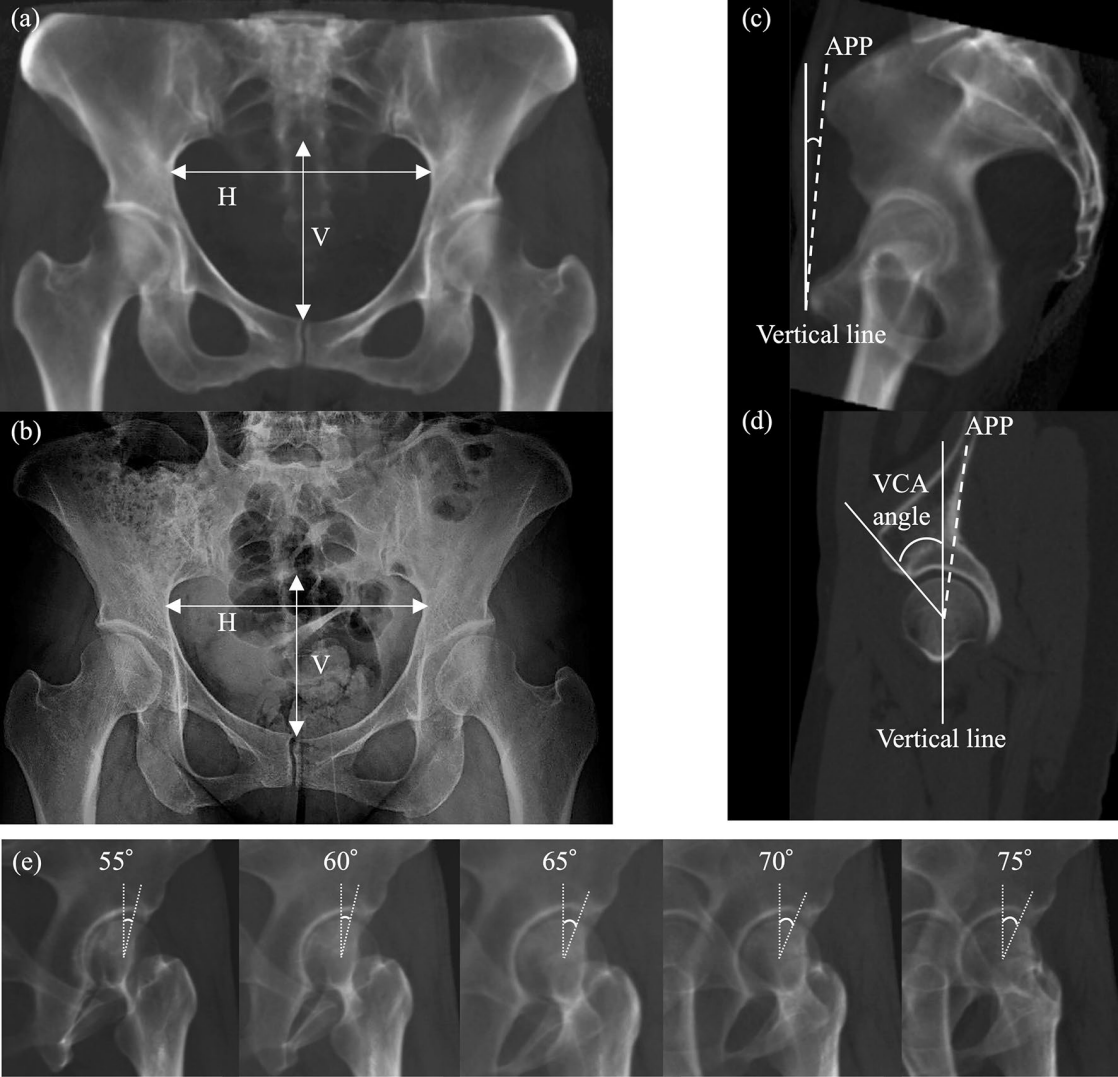
DRR image (**a**) and standing radiograph (**b**). Adjust the pelvic inclination (**c**) so that the vertical-to-horizontal ratio of the pelvic foramen is the same between (**a**) and (**b**). The arrows indicate the vertical distance between the bilateral sacroiliac joints and pubic symphysis and the maximum horizontal diameter of the pelvic foramen. The pelvic inclination was defined as the angle between the solid line (z-axis) and the dotted line (APP). (**d**) VCA angle was measured as the angle between the vertical line and a line connecting the femoral head center and the anterior end of the acetabulum after adjustment to the standing position. The dotted line indicates APP. (**e**) DRR image of the FP radiograph (rotating AP image 65° in the axial plane), ± 5° and ± 10°relative to the FP radiograph (rotating AP image 55°, 60°, 70°, 75°in the axial plane). Indicates a change of sourcil edge with pelvic rotation. The ACEA was measured for each DRR image. *DRR* Digitally reconstructed radiography, *APP* Anterior pelvic plane, *VCA* Vertical-center-anterior, *FP* False profile, *AP* Anterior–posterior, *ACEA* Anterior center edge angle.

The DICOM datasets were imported into 3D planning software (3D template; Kyocera, Osaka, Japan). First, the pelvic 3D coordinate system was defined based on the anterior pelvic plane (APP)^11,23^. The pubic tuberosity was defined as the origin, and the APP, including the pubic tuberosity and bilateral anterior superior iliac spines, was defined as the coronal plane. Next, the pelvis was realigned in the sagittal plane to simulate patient-specific pelvic inclination in the standing position. Digitally reconstructed coronal radiographs considering the patient-specific standing positions were created so that the vertical-to-horizontal ratio of the pelvic foramen matched the standing radiograph by incrementally adjusting the projection angle^11,23,24^ (Fig. 3a, b). Changes in sagittal pelvic inclination in the standing position relative to the APP were measured, and a positive value indicated anterior pelvic inclination (Fig. 3c).

### Evaluation of anterior acetabular coverage in the CT sagittal plane

After adjustment to the patient-specific standing position, the anterior acetabular coverage in the CT sagittal plane was measured as the angle between (1) the line connecting the center of the femoral head and the anterior end of the acetabular roof and (2) the line from the center of the femoral head vertically^25^. This angle was defined as the vertical-center-anterior (VCA) angle in the sagittal CT plane (Fig. 3d). According to a previous study^12^, a VCA angle < 32° in the sagittal CT plane after adjustment to the standing position is defined as inadequate anterior coverage.

### DRR image reconstruction and measurement of the center–edge angle

Consequently, the DRR image of the FP radiograph was reconstructed by rotating the AP image in the standing position to 65° in the axial plane. This DRR image was defined as an FP radiograph with neutral rotation (NR). Then, DRR images rotated by ± 5° and ± 10° in the axial plane relative to the neutral FP radiographs (at 55°, 60°, 70°, and 75° from the AP view; Fig. 3e) were reconstructed. The positive value of pelvic rotation was defined as rotation toward the affected side, approaching a true lateral view. Finally, various pelvic inclination was simulated. DRR images of each pelvic inclination were reconstructed by tilting the pelvis ± 5° and ± 10° from the patient-specific standing position and then rotating the pelvis 65° in the axial plane. The positive value of pelvic inclination was defined as an anterior pelvic inclination. The ACEA was measured for each DRR image. The ACEA is defined as the angle between (1) the line from the center of the femoral head, perpendicular to the transverse axis of the pelvis, and (2) the line from the center of the femoral head to the most anterior edge of the acetabular sourcil^2,17,26^.

### Examination of the “appropriate range of positioning”

Detailed validation of the “appropriate range of positioning” criteria^17^ was performed. In each DRR image, the diameter of the femoral head and the horizontal distance between the medial aspects of the femoral heads were measured. The distance-to-diameter ratio, which is the ratio of the horizontal distance to the femoral head diameter, was calculated according to a previous study^17^. Pelvic rotation was simulated in increments to determine the axial rotation angle corresponding to distance-to-diameter ratios of 0.67 and 1.0.

### Statistical analysis

Data analysis of the ACEA measurement for each DRR image in different pelvic rotations was conducted using repeated-measures analysis of variance. Correlations between the VCA angle in the CT sagittal plane (after adjustment to the standing position) and ACEA with NR were investigated using Pearson’s correlation coefficient analysis, and the reference value of ACEA with inadequate anterior coverage (VCA angle < 32°) was investigated by receiver operating characteristic (ROC) analysis. Statistical analyses were performed using JMP statistical analysis software (version 15.0; SAS Institute, Cary, NC, USA), and statistical significance was set at *p *< 0.05. To evaluate intra-observer and interobserver reproducibility, measurements were performed twice by one examiner (Y.K.) and once by another examiner (S.K.) in the study group. The intraclass correlation coefficient and the interclass correlation coefficient were good 0.89 to 0.97 and 0.84 to 0.94, respectively) for all measurements.

### Ethics approval and consent to participate

This retrospective study was approved by Kyushu University institutional review board for clinical research (No. 30-137) and was conducted in accordance with the Declaration of Helsinki. Written informed consent was obtained from all patients prior to the study.

## Results

**Figure 4:**
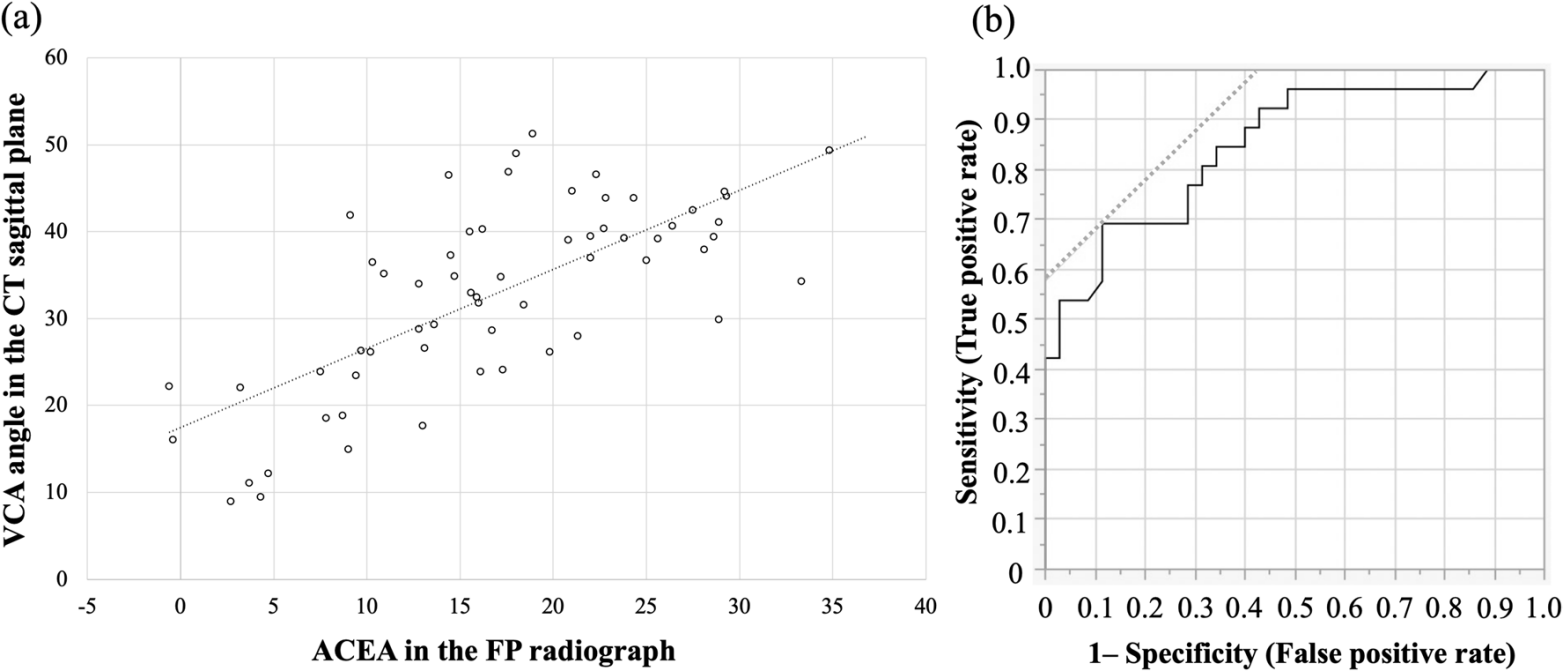
(**a**) Correlations among the VCA angle in the CT sagittal plane and the ACEA on the FP radiograph with neutral rotation. (**b**) The receiver operating characteristic curve for inadequate anterior coverage with the ACEA in FP radiograph. The cut-off value of the ACEA was 13.6° (sensitivity 69%, specificity 89%, area under the curve 0.85). *VCA* Vertical-center-anterior, *ACEA* Anterior center edge angle, *FP* False profile.

Patient demographics and radiographic data are shown in Table 1. The standing pelvic inclination was -0.24°. After adjustment to the standing position, the VCA angle in the sagittal CT plane was 32.8° on average, of which 26 hips (43%) had a VCA angle < 32°. The ACEA on the FP radiographs with NR was 16.8° on average, which showed a good correlation (r = 0.71, *p *< 0.001) with the VCA angle in the CT sagittal plane after adjustment to the standing position (Fig. 4a). Receiver operating characteristic (ROC) curves indicated that an ACEA with NR < 13.6° was associated with inadequate anterior coverage (sensitivity 69%, specificity 89%, area under the curve 0.85) (Fig. 4b).

**Table 1:** Patient demographic and radiographic data.

Parameters	n = 61 hips
Age (year)	40.2 ± 10.6
Sex	Male: 4, Female: 57
Height (cm)	158.6 ± 5.9
Body weight (kg)	56.4 ± 10.6
BMI (kg/m^2^)	22.3 ± 3.7
LCEA (degree)	12.3 ± 6.4
Tönnis angle (degree)	19.9 ± 5.4
Sharp angle (degree)	47.3 ± 3.5
Standing pelvic inclination (degree)	− 0.24 ± 6.9
VCA angle in the CT sagittal plane (degree)	32.8 ± 10.7

Values are given as the mean and standard deviation.

*BMI* Body mass index, *LCEA* Lateral center edge angle, *VCA* Vertical-center-anterior.

**Figure 5:**
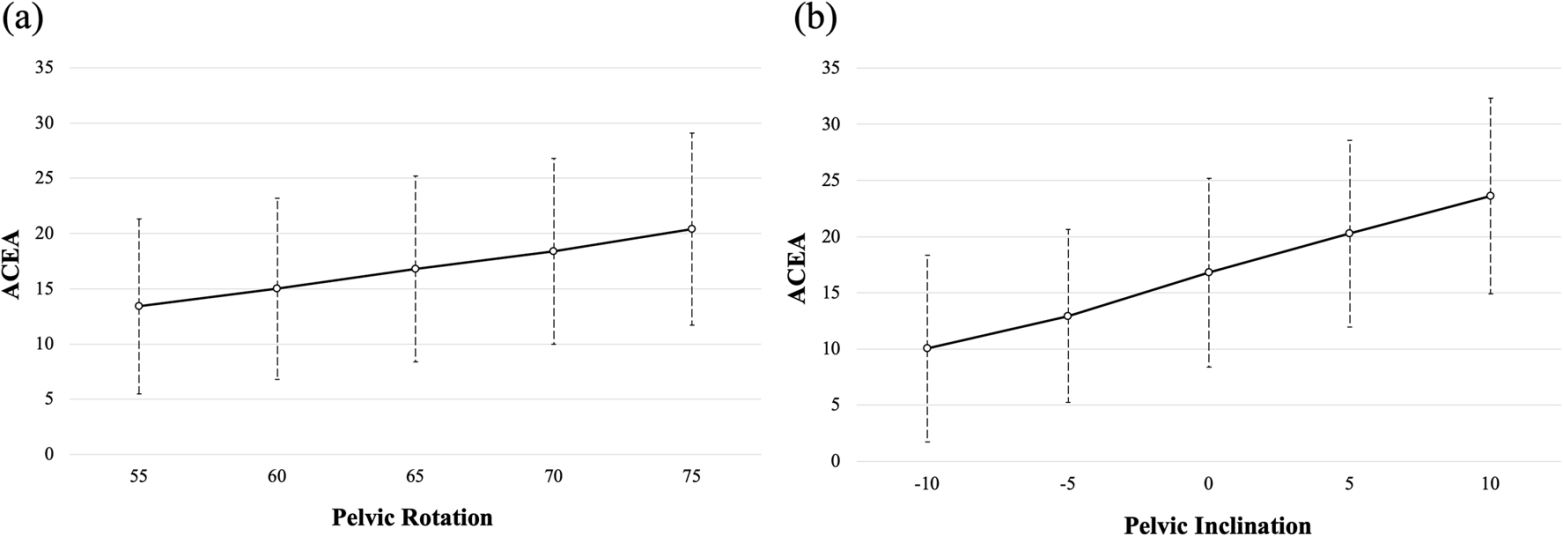
(**a**) The line plots show the values of ACEA (mean and standard deviation) for each pelvic rotation. (**b**) The line plots show the values of ACEA (mean and standard deviation) for each pelvic inclination. The positive value of pelvic inclination was defined as an anterior pelvic inclination. *ACEA* Anterior center edge angle.

**Figure 6:**
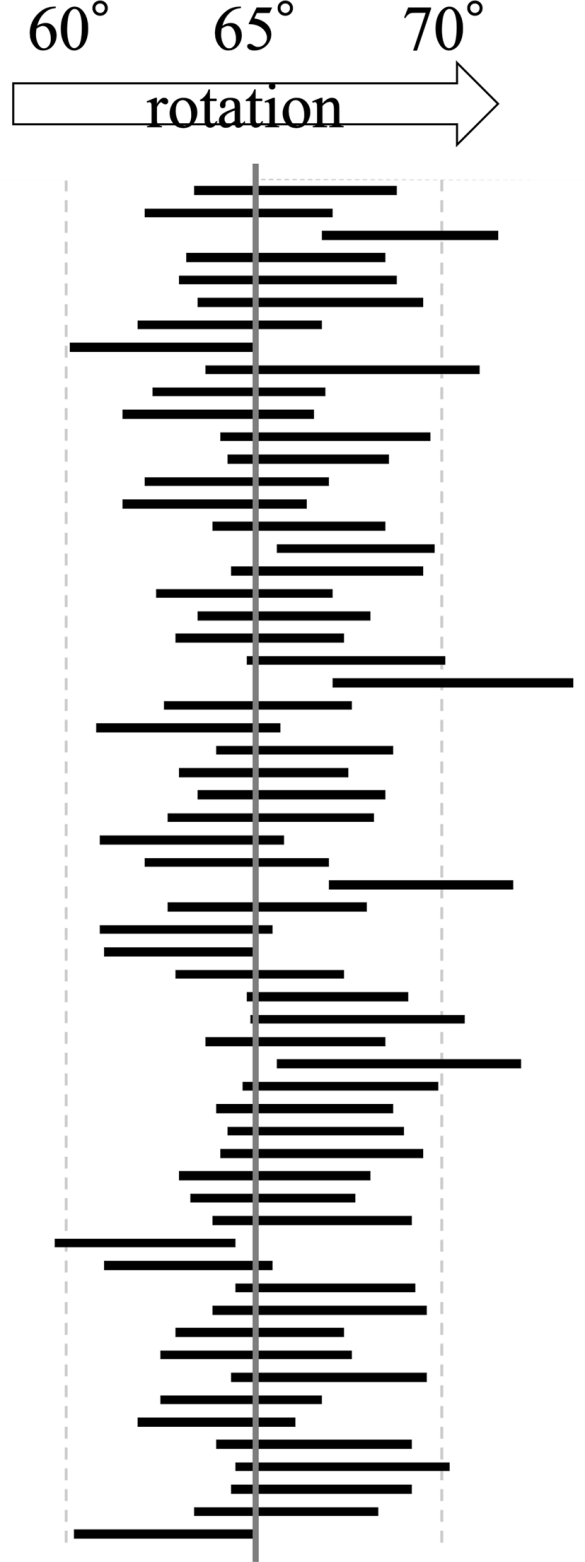
Distribution of the “appropriate range of positioning”. The horizontal bars indicate the “appropriate range of positioning” in each case. In 55 hips (90.2%), pelvic rotation of 65°, indicated by the central vertical line, was included in the “appropriate range of positioning”.

In the simulation of pelvic rotation, the ACEA measurement increased by 0.35° for every 1° increment approaching the true lateral view (Fig. 5a). In the simulation of pelvic inclination, the ACEA measurement increased by 0.68° for every 1° increment of anterior inclination (Fig. 5b). The rotational pelvic positions that met the “appropriate range of positioning” (0.67 < distance-to-diameter ratio < 1.0) were found in the range of 5.0° between 63.3° and 68.3°. Pelvic rotation for all patients meeting the "appropriate range of positioning" criteria were shown in Fig. 6. Fifty-five hips (90.2%) met the “appropriate range of positioning” criteria for the FP radiographs with NR.

## Discussion

The present study showed a good correlation (r = 0.71) between the ACEA on the FP radiographs and the VCA angle in the CT sagittal plane after adjustment to the patient-specific standing position. In contrast, a previous study reported no significant correlation between ACEA on the FP radiograph and anterior acetabular coverage in the CT sagittal plane^25^. This inconsistency can be explained by the fact that the previous studies did not consider patients-specific pelvic inclination in the standing position and that the pelvic rotation was not strictly adjusted during radiography. In the present study, a good correlation was found by precisely matching the pelvic rotation and inclination. Although the FP radiographs are useful for diagnosing dysplasia and detecting the joint space^15^, studies reporting valid reference values of ACEA for preoperative planning for PAO have been scarce. Preoperative planning requires evaluation of the anterior acetabular coverage. Kitamura et al. performed virtual PAO and developed finite-element models to simulate joint contact pressure^12^. Although normal joint contact pressure was achieved in 63% of patients by lateral acetabular rotation in that study, the anterior rotation should be additionally considered in patients with a VCA < 32° in the CT sagittal plane after adjustment to the standing position. In the present study, ROC curve analysis showed that patients with an ACEA < 13.6° on FP radiographs had low anterior coverage corresponding to < 32° on sagittal CT, with a good area under the curve, sensitivity, and specificity. This reference value can evaluate anterior acetabular coverage on standing radiographs. Moreover, CT is not routinely performed for preoperative planning in all institutions because of the high radiation dose and cost. For patients with ACEA less than 13.6° on FP radiographs, detailed preoperative planning using CT images is recommended for three-dimensional acetabular correction. To the best of our knowledge, this is the first study to set a reference value for ACEA in FP radiographs for preoperative planning of PAO taking the standing condition into account.

As pelvic rotation increased (approaching a true lateral view), the more anterior part of the acetabular rim is projected as the sourcil edge^27^ (Fig. 3e), and the ACEA increased by 0.35° for every degree of increased rotation. This effect was also observed in previous studies. Ryan et al. conducted a study on the effect of pelvic rotation and reported an approximately 0.3° change in ACEA with every degree of rotation in 11 cadaver pelvises^17^. Putnam et al. reported changes in ACEA of 0.18° with every degree of rotation on eight hips of four cadaveric pelvises^18^. Previous studies included cadavers without dysplasia, whereas the current study is the first to investigate pelvic dysplasia. Compared to the current study, the slight difference in values in the previous studies may be due to the type of study subjects enrolled. However, as pelvic inclination increased (anterior inclination), the ACEA measurement increased by 0.68° for every 1° of anterior inclination. Putnam et al. also reported changes in ACEA of 0.65° for 1° inclination and that the FP radiograph is a standing radiograph, wherein patients were instructed not to assume a forced posture during the radiographic examination^18^. The radiographs also ensure that the patient's anatomy is functionally represented and minimize the influence of inclination on the ACEA measurements. The patient should be positioned in a natural standing position so that the radiograph accurately represents the patient's anatomy.

The “appropriate range of positioning” criterion from Lequesne and de Seze has been conventionally used to exclude inappropriate images. Putnam first examined this criterion using eight cadaver hips^18^. The position was rotated by 5°, and the measurements were repeated to validate the criterion. They reported that all cases met the criteria with an obliquity between 60° and 70°. The criterion was fulfilled in 55 hips (90.2%) of the 61 hips of FP radiographs taken at the 65° oblique position. The criteria reported by Lequesne and de Seze may be useful for selecting inappropriate images. Whereas previous reports used a small number of cadavers for validation, in the present study, we performed further simulations using a large number of CT images to determine the rotation range that met the criteria in each case. The results showed that the average optimal range was 5.0° (63.3–68.3°). Therefore, when evaluating an image with the “appropriate range of positioning” it is necessary to assume a variation of 1.7° (0.35 × 5) due to pelvic rotation.

This study had limitations. First, this study population was limited to Japanese patients. Second, most patients in the study population were females. Female patients are reported to have more acetabular dysplasia than male patients^28^. However, Japanese patients with hip osteoarthritis have been reported to differ from Caucasians in terms of sex distribution and frequency of acetabular dysplasia as an etiology^29^. The results may therefore not be generalizable to different races. Third, in patients with severe acetabular dysplasia, when LCEA < 0°, the VCA angle cannot be measured because the acetabulum cannot contact the femoral head in the CT sagittal plane through the center of the femoral head. Previous reports have treated these cases by assuming anterior hip coverage of 0°^30^ or excluded these cases^31^. Although none of the patients in this study had such severe acetabular dysplasia (LCEA < 0°), our results may not apply to patients with such severe acetabular dysplasia.

In conclusion, during preoperative planning of PAO, an ACEA < 13.6° on FP radiographs indicates insufficient anterior acetabular coverage. Images with the “appropriate range of positioning” can also have a measurement error of 1.7° due to pelvic rotation. Therefore, when evaluating radiographs, it is important to note these measurement errors.

## Data Availability

The datasets used and/or analyzed during the current study are available from the corresponding author on reasonable request.
